# Potentiation Makes the Medicine Homœopathic

**Published:** 1882-10

**Authors:** B. Fincke

**Affiliations:** Brooklyn, N. Y.


### POTENTIATION MAKES THE MEDICINE HOMŒOPATHIC.

B. FINCKE, M. D., BROOKLYN, N. Y.

### I.

"Hahnemann geh' du voran
Du hast die grossen Wasserstiefeln an."—*Saxon proverb.*

This sentence is only a different rendering of the thesis, "*The homœopathic dose is infinitesimal,*" which was presented to the New York State Homœopathic Society, February 14th, 1865, and the manuscript of which was returned to me with scorn by the then secretary, Dr. Horace M. Paine, who wrote on the back of it, "superlative nonsense, p. 11, 12." Looking at these pages, I was much surprised to find that they contained quotations from Hahnemann's own works from the years 1799 and 1801, and I must confess that this observation comforted me not a little in my chagrin, at being repulsed in such an ignominious manner. This article, however, was afterwards printed as an appendix to my essay on *Homœopathics and High Potencies*, Phila., A. J. Tafel, 1865, and is now recommended for perusal in arriving at a correct judgment about the posological question which has set friends and foes by their ears indiscriminately, though quite unnecessarily, because it has been settled and closed long ago, when Hahnemann declared: "I hold none to be my follower who, besides an irreproachable, truly moral conduct, does not exercise the new art at least in such a manner, that his remedy given to the patient contains in an unmedicinal vehicle (sugar of milk, or watered alcohol) the medicine in such a minute dose, that neither the senses nor chemical analysis could discover in it the least absolutely injurious medicament, even not at all the least properly medicinal thing; all of which presupposes a minuteness of dose, which, beyond contradiction, makes all apprehension of every medical state superintendence disappear."—(Kleine Schriften, ed. Stapf. vol. ii., p. 199.) But, if the champion of Homœopathy, who, of late, has astonished the homœopathic world with his experiments in the use of the highest potencies of his own make, and yet declares: "As a homœopathician, we consider ourselves bound solely by the law of the similars, and not by the dose, or by the dynamization of the remedy," if Dr. Skinner coincides with the arch-enemy of high potencies, Dr. H. M. Paine, who deems "the theory of dynamization to be essentially non-homœopathic," in that there is no need of potentiation to make a medicine homœopathic, there must be some screw loose in regard to a correct understanding of fundamental facts, and the following is offered as a means to clear up that misapprehension, rather than to pour oil into a fire which threatens to consume all the laborious endeavors of many good and intelligent men to uphold the cause of Homœopathics as the science and art of medicine.

Potentiation, in the beginning, has not been a matter of theory, but of practice. When Hahnemann perceived that his large doses of Veratrum in the famous colicodynia case almost killed his patient, he commenced to lessen his doses. It was simply an element of induction, namely, his observation and experiment which gradually led him on to the astounding fact, that by lessening the doses, more curative forces would be developed from the original medicine employed than had been observable in its crude state. This is simple matter of fact, and no sleight-of-hand can substitute for it the idea, that theory and speculation led to the discovery of potentiation. Finally, Hahnemann arrived at the certainty of medicinal action, both in sickening and healing in even as high as a thirtieth centesimal potency. This tremendous fact was thrown into the teeth of the whole medical world by Hahnemann, with a courage rarely equaled. It has been so often used to open people's eyes as to the folly of trusting to such ridiculous remedies, so-called, but actual nothings, for the manufacture of which, however, large orbs of vehicle like Siriuses, would not suffice, that in turn, it may be used in truth to open people's eyes as to the wonders which do never cease to appear, perhaps, for a double purpose: first for the benefit of the human race and second for the discomfiture of the learned tribe. For this potentiation, upon which Hahnemann stumbled in his earnest search for efficient weapons to conquer disease, is a wonder which no human understanding can explain nor fathom. All we have to do, is to accept the fact, that by potentiation medicine is made more curative than it is in the crude state. What we want to know of medicine, treated with large masses of vehicle in order to distribute it throughout its bulk so that every particle will be endowed with the medicinal force of sickening and healing—is only, that its force can be observed clearly and undoubtedly in its application to the human and animal organism in health and disease. This force, then, is a potency which by no other means can be shown to exist, than by the finest re-agent the world knows, the human organism, and for this reason Hahnemann very aptly called the medicine thus treated *Potency*, and the process by which it is obtained *Potentiation*. Now, by the homœopathic argument it is evident, that like cures like, because it causes like affections, and from this the advocates of crude drugs and low potencies claim, that the quantity of medicine of the dose has nothing to do with the homœopathic law, and everybody may do just as he likes. But even the ground-rule like to like should teach them that they do not act according to it when they do just as they like, because there is no liking allowed to the physician in the relationship of medicine to organism. There are definite proportions in which the medicine stands to the organism whether sick or well, and it is for the homœopathician to find out these proportions, or else as physician he fails in his object to cure, and is deficient in his vocation. The relation of the crude drug to the system is different from that of a five-millionth centesimal potency of it. But this being thought too transcendental, because it transcends the experience of the many, let us go back to where Hahnemann left it when he published the fifth edition of his *Organon* of the healing art, *i. e.*, to the thirtieth centesimal. This thirtieth is already infinitesimal as a fact if we exclude the latest discoveries of Neural Analysis in its chronoscopical and electro-magnetic methods which, indeed, assign positive values, the first to a potency as high as the four thousandth centesimal, the latter as high as the five millionth, centesimally. But let us take the thirtieth of Hahnemann to be infinitesimal, as of old, for the benefit of those who have not yet succeeded in getting out of the slough of despond, the lower skepsis.

Take a drop of hydrocyanic acid and compare it with the thirtieth potency of it. Would it not be criminal in any physician, even a homœopathist, who does just as he likes with regard to the dose, to give to his patient a drop of that poisonous acid? Of course, he would be hung for it by his neck. Just so the principle to do just as one likes, without principle, ought to be hung up by its neck, because it murders all intelligence derived from the scientific instrument of Induction. But the homœopathician wants to apply this poison which he supposes, for the very power of it for killing, to be a powerful curative if given in the proper dose. In order to find this out, the acid must be treated with an inert vehicle in order to lessen its deleterious effects, and to develop its healing forces. This is potentiation. For on proceeding with it to the thirtieth centesimal potency, Hahnemann finds that the acid lost all its poisoning and acquired only curative and morbific powers, for, when applying it to a suitable human organism, it produces a string of symptoms which, then, are found to form its remedy for another human organism which presents similar symptoms of sickness. What then has happened to the medicine treated by potentiation? It has been made homœopathic by potentiation, because in its crude state it had not been homœopathic at all, it had no pathic relation to the organism, only a toxic one, and this pathic relation has been developed only after submitting the medicine to the process of potentiation.

Take from the opposite range of medicines, Silicea. One single grain of it answering the amount of one drop of hydrocyanic acid in the above instance. What would it do in the hands of any physician, even of a homœopathist, who does just as he likes? Nothing. It would be perfectly inert in his hands whether he would apply it to a healthy or sick organism of even great sensitiveness (except, perhaps, to a Caspar Hauser). Therefore, the substance would be rejected as being entirely unfit as a remedy. But what does Hahnemann do? He adopts the rejected child, and nurses it into homœopathic life by potentiation, and lo! when he arrives at the thirtieth potency, one of the most powerful polychrests has been developed, without which no homœopathician could do in some of the most loathsome diseases of mankind. Here is the crude drug which has no medicinal action whatever in its crude state, therefore no application in medicine because it has no relation to the organism, there being no medical data to go upon. But Hahnemann, with his keen insight, found the way to render the inert drug active in the homœopathic sense, and therefore, the potentiation has made the substance homœopathic in the thirtieth potency.

What has been predicated here about the thirtieth potency, applies to any higher potencies, too. If his followers have carried the numbers higher than Hahnemann did, they have done little more than followed in his track. And anybody meditating upon the efficacy of even the thirtieth potency will not find it difficult to extend the series of potencies to any extent in his practice, because this is only matter of fact and experiment, and by no means of theory and speculation.

### II.

But, granted all this to be as here demonstrated, how is it that so many cures effected by the majority of the homœopathic physicians are wrought by low potencies, and even crude drugs? These gentlemen are entitled to the same credit for their statements as those in the minority; they express themselves everywhere highly satisfied with their success, and if it were for them Hahnemann need not have been in existence, so far as posology is concerned. They, then, reason from their experience that Hahnemann was correct in the beginning of his career, when he applied large doses and degenerated later, when he recommended the infinitesimal dose as the homœopathic dose. But as far as Hahnemann's intellect is concerned, it could not possibly have suffered from senility when it gave to the world the *Materia Medica Pura*, which was commenced at the ripe age of fifty-six years, and long before Hahnemann had advocated the infinitesimal dose. And there is a fatal defect in the reasoning of the adherents of low potencies, that they only speculate upon the nature and the effects of high potencies, but do not apply the only test which alone can lead to the solution of the posological question, viz., the experiment. The statements of their cures with low potencies and crude drugs cannot be doubted. Nay, those physicians who in their practice are in the habit of applying high potencies, have their own experience about the efficacy of low potencies and crude drugs, derived from experiments in their practice, and they do not say that these drugs have proved inert or deleterious in all cases, but in many cases they have proved curative, but, after rising in the scale, they prefer the higher potencies, because their action is quicker, safer and more enduring. They even do not deny themselves the right, in certain cases, "where they see fit," to apply the homœopathic remedy in a large dose and low potency, because they are also entitled to this privilege by their parchment; but, after all, these are very exceptional cases with them. This procedure on the part of the minority, first to test the lower and then the higher potencies, in order to find out in which the lower and in which the higher might be preferable, is worthy of all praise, and must be recommended to the majority, who claims indeed the whole scale of potencies, but in fact rarely rises beyond the first decimals and centesimals, and perhaps the thirtieth in chronic cases. Before, then, these gentlemen of the low persuasion do not faithfully try what the high potencies may be able to accomplish, they should be silent upon that topic, because it would be clearly against the scientific requirement of intelligent action, viz., induction, to talk of things which they do not understand.

But inasmuch as the minority advocating the higher potencies are perfectly cognizant, that also cures are perfected by low potencies and crude drugs, this circumstance must be looked at in the light of potentiation, such as Hahnemann has taught us, aside from the predilection of the majority for large doses.

There is no potentiation yet brought to bear upon a crude drug such as when Hahnemann applied a drop of the fresh Bryonia juice in the celebrated case of the washerwoman's rheumatism, and yet it had the most desirable effect of a perfect cure. Are not by this simple fact all high-potentialists beaten to pieces? What, then, is the use of the tremendous labor to carry up to the hundred thousand or even to millions, about four or five hundred remedies of the homœopathic materia medica? Had we not better be content, and abide by the first doings of Hahnemann when he was so successful? Thus talks the majority. Yes; indeed, the advocates of the low practice are to the present day on the standpoint of Hahnemann, some seventy years ago. Judging, then, from their progress in this matter, it will, in the most favorable case, take them seventy years more only to arrive where Hahnemann was in 1810, when he published the *Organon* for the first time, if not the impending electric era will help them along quicker. But that would not explain why a large unpotentiated dose may also cure.

To commence with the beginning, as a general rule a drug positively poisonous and endangering life, should never be used as a remedy in a large dose and crude condition; it involves a criminal action.

Next comes the class of drugs which do not act toxically in every instance, but only in certain individuals and diseases. Here appears an important element, viz., the susceptibility of the subject. Some people are more affected by some drugs, some less. Some bear enormous doses in certain diseased conditions, whilst others succumb under even moderately large doses. Therefore the susceptibility of the subject must be estimated and the dose accommodated to its wants. If such is correctly done, then a cure may be effected even with larger doses. But the estimate of the actual susceptibility requires many acquirements which at the present stage of knowledge the physician cannot have. He guesses. If he guesses right the patient may recover; if not, he will die. Such an alternative no honest legislator should admit in the community. The art of healing should always keep within the bounds of safety for the patient, if not for the physician, as a general rule. There are cases in surgery, including the tocological part of it, when an operation is necessary to save a patient's life, whilst at the same time it may be endangering it on account of unforeseen and incalculable circumstances. But this has nothing to do with therapeutics, where "*salus ægrotli suprema lex.*" Wherever life is jeopardized by dangerous medication, it is at the risk of the attending physician, and it should not be encouraged for imitation by the profession, especially if it can be shown that there are safe and gentle means within the range of the homœopathician which make such performance unnecessary.

Still, there are cases which undoubtedly get well by large doses, which in other cases would prove fatal; and this stumbling-block in the way of the general use of high potencies should, if possible, be removed, so that the many who are deterred by it see their way clear. There may be various probabilities which may explain what happens when a large dose cures. In the first place, the substance may not be suscepted in its whole quantity, because the organs of the subject are not sensitive enough to be affected by it. Second, the substance may only partly be suscepted, and act according to its homœopathicity sufficient to cure the subject, whilst the remainder of it is carried off by the *primæ viæ,* without entering the system at all; or, third, it may be counteracted by the force of the organism rising to an equal degree of power as the drug is able to exert and neutralizing the drug substance in some manner, and making it inert. And there may be other modes of operation by which a cure is facilitated and the danger checked; or, fourth, the substance may act as a nutritive substance, taken up for sustenance and repair of the body. This would seem to be a hygienic measure, which recommends itself at first sight, on account of its physiological plausibility. But inasmuch as these substances are powerful remedies, able to make well people sick, their application in large doses should be discarded on homœopathic principles. A single instance will show the reason. Calcarea phosphorica has been used to facilitate the union of broken bones, and in one case of an old man with broken thigh-bone (Hering, *The Twelve Tissue Remedies:* Bœricke & Tafel, 3d ed., p. 5), with such an effect that the callus contained fifty to sixty times more phosphate of lime than had been taken. Hering judges that the phosphate of lime given as a nutritive remedy had acted as a functional one. If so, it was nutritive and functional at the same time. In another case, given to me by my friend, Dr. John C. Robert, where an old man æt. eighty years had broken the neck of his right femur, the bones hesitated to unite, under constant pain and sleeplessness. Symphytum officinale 10 m (F.) was given, one dose, January 18th, 6 P. M. The pain subsided immediately, but patient was raving in his sleep, ordering men about, wanted cobwebs swept off the walls, saw a crucifix moving about the wall, complained of working too hard. Terrible pain in the thorax on the right side, that he could not draw his breath, obliged to hold the part with his hands; relieved by Acon Cm (F.); pulse intermittent every fourth beat, small, thin. January 20th, 10 A. M., ℞ Symphytum officinale 10 m (F.) in half a tumbler of water, a teaspoonful every two hours. After that he commenced raving as before, with the very same fancies, so that his relatives became afraid and called the priest, who gave him the last rites of the church. But the same evening patient was sensible again, and the next morning everthing was in good order, pulse regular, mind perfectly sound, and he could move himself in bed. All along the fracture had improved, and then he got well without further trouble. Now, was not this high potency of Symphytum just as well a nutritive remedy in uniting the broken bone as a functional one in producing the necessary assimilation of the bony matter? But there is this difference. In the phosphate of lime case the nutritive action produced a callus which contained fifty to sixty times more phosphate of lime than had been given. From what source did the system get all that large amount of phosphate of lime? The large doses must have forced it to deposit it from other sources of assimilation which are not intimated, and the callus must have been more brittle than before, which was certainly no desirable effect in the old man, because it must have been liable to be broken again at the next slight occasion. But in the Symphytum case there was the organismal natural assimilation taking place, induced by the remedy acting in virtue of its homœopathicity upon the nerve-centre, which on its own account, after having received the impetus from the remedy, instituted the restoring and healing process, ending in the union of the broken bone. Still, here also a surplus is recognizable, but it is only a passing pathopoetic action, which serves in the future as a good proving, without having subjected the patient to any but very temporary harm.

Thus this division of remedies into functional and nutritive ones, under the head of tissue-remedies, does not find a fit place in Homœopathy, though the single facts of how they act pathopoetically upon the system, will find their appropriate registration in the *Materia Medica Pura.*

Hering, himself, who evidently is the father of this new departure of Schuessler, who followed in the wake of Grauvogl, gives the criterion of this view in the following words (*u. s.*, p. 18): "Such chemicals as have a function in certain tissues of the body, are in diseases of such tissues when given as a nutritive, the better equalizers of the disturbed state of the functions," but he adds, warily, "Of course they are brought in a molecular state," which means the more highly they are potentiated. And with that Hering puts himself in the right, whilst Grauvogl and Schuessler are in the wrong.

The homœopathic high potency, well selected according to symtoms-simility, and in proportionate dose, will act as a nutritive if nutrition is at fault, and it will restore the function when that is out of order. So there is no need of Homœopathy made easy by the new system of Schuessler, though gratefully we assimilate what there is good in it.

Of all these probabilities, the one which allows only a few infinitesimals to act upon the nervous system by virtue of its homœopathicity, whilst the rest of it goes the way of the flesh without exerting any action at all, will be found the most reasonable, because a few infinitesimals of the same medicine, obtained by potentiation, will do the very same thing, *i. e.*, effect a cure if the remedy has been selected homœopathically correct. The remedy, generally, is taken by the mouth. As soon as it passes the tongue, the nerve-papillæ, by natural selection, which is paramount to the homœopathicity of the drug in the organism, suscept the curative quality and propagate it by nervous force to the nerve-centre, where the cure is wrought by restoring the equilibrium changed by disease, and from there, healing the affected parts. This susception occurs in the short time of a minute or two, and less, as can be shown by the electro-magnetic method of Neural Analysis, before the drug enters the stomach. What changes the medicine will work in the stomach, if that is the *locus suscipiendi*, is uncertain; this depends upon chemical processes, which cannot be said to be sufficiently well understood. The probability is, that most of the medicine leaves the system by the alimentary canal, or by the urinary or other emunctory organs, or is retained in the tissues, making them inert by envelopment, without exerting any deleterious action. But it may also accompany the chyle in its transformation into blood, or enter directly the venous system, and then is supposed to act by the intervenience of the circulation upon the diseased parts of the organism. This may be so, but what an uncertain, roundabout way it is! Just so with the hypodermic injection. The drug is supposed to be carried by the circulation to the ailing part, and to act, by entering the capillaries, directly upon the nerve-terminations. But how to prove that the supposition is true? True it is, that the skin-tissue, with its fine nerve-terminations and capillaries, is torn by force, and the drug substance comes just as well in immediate contact with the nerve-matter as with the blood. Is it probable that the sensitive nerve-substance will wait for the action from the capillaries upon the nerves, in order to be affected? No, it received the primary impulse, and carries it according to the homœopathicity of the drug, to where it belongs. But does not the hypodermic injection deaden the pain at the circumference where it is made? Yes, but not that alone; before that it propagated its deleterious influence to the nerve-centre, in the brain, and forces it to react upon the unscientific inroad upon its domain, and thereby weakens its central force, as now is well proven by the adversaries of this surgical interference with medical matters. The same fallacy appears in the way vaccination is looked at by the old school, as the advocate of large doses. Says the sagacious Hahnemann, in his *Chronic Diseases*, vol. i: "If the cow-pox catches, it happens in that moment, when, on inoculation of the same, the morbid fluid comes in contact with the exposed nerve in the bloody scratch of the skin, which then, in the same moment, irrevocably communicates the disease dynamically, to the vital force (to the whole nervous system)." Then the organism, in self-defense, produces the specific disease depending upon the introduction of the virus. If, then, as there is no doubt, it is the mere contact of the virus with the cutaneous nerves, scratched open for its insertion, which is essential for the vaccination and for the protection by it, it is claimed that this object is gained in a much safer and pleasanter manner, not by poisoning the system with the crude virus, and questionable virus at that, but by taking a high potency of the very virus we want to counteract in the future, upon the tongue in the same manner as we take the remedies for proving. If there is any susceptibility to this disease in the system, which we want to annihilate, it will show itself in a complex of symptoms which will be more or less characteristic of the small-pox-disease, and pass off without any inconvenience whatever, after a little while. There is no reason why this procedure should not protect from this loathsome disease any less than the common operation of vaccination, which, after all, is only a crude homœopathic measure, and, therefore, is to be repudiated, because it so often fails of its object and adds more misery to that already existing.

Large doses, then, may cure, and potentiation be out of question altogether in such a cure, but it certainly is a very uncertain, and often dangerous, therapeutic proceeding, which has no claim to science nor art, and follows a crude empiricism, which was to be excused in olden times, when people did not know any better, but since Hahnemann's time, nobody should fall back into that antiquated medication, endangering and shortening so many valuable lives. For the real cures with large doses are very few, and they are dearly bought with many failures. It also should not be forgotten that the bulk of physicians is not of the high order, as the heads of the profession, and that many have not the discriminating intellect to apply a dangerous remedy in the right place. The Schoenleins, the Oppolzers and the Clarks are very few who may be trusted, but the many should be prevented by proper scientific rules, to wallow in the entrails of the people whose welfare is confided to their care. This cannot be done by legislation, but only by the enlightenment which science is able to give, so that every thinking physician will clearly see that it is not necessary to administer large doses, even in dangerous cases, because Hahnemann has first taught us to prepare medicine in such a manner, that it exerts the powerful action of large doses without endangering the life and comfort of the patient.

But our homœopathic brethren of the lower persuasion, do not like to be ranged among those physicians, who, by large doses, endanger their patients. They confine themselves to the lower decimals and centesimals, and only occasionally, when they see fit, claim the right to use as large doses as are used in the allopathic treatment, if their low potencies will not answer. It is those homœopathists whom Hering meant, when he termed them those "who stand half-way." It is those whom Dunham went to succor in his famous address, in which he advocated "liberty of medical opinion and action." But all this indulgent aid of well-meaning homœopathic brethren, is of no avail, and actually has done more harm than good, because these half-way men cannot see their way clear. There is always that stumbling-block of the large dose which cures, lying in their way. If the large dose cures, why take a small dose? Why quarrel about the dose at all? In *omnibus charitas!* Let us unite upon the *similia similibus curantur* of Hahnemann, which we acknowledge to be a good rule to go by, except where it is not universal enough, and let everybody do about the dose as he pleases. We don't want any popes, nor any bosses, we want to be every one his own pope and boss. This, indeed, is the gist of the matter, and what it all comes to, if there is no guiding star at the north-pole.

### III.

This guiding star, what else can it be than Hahnemann's *similia similibus curantur?* If we had no scientific foundation for it, which we have, for it coincides with the first laws of motion and even leads to the universal principle of Homœosis underlying them (see *High Potencies and Homœopathics.* Tafel, 1865), our cures and our provings would show the truth of it in the records of innumerable cases of experience and experiment. There has been no scientific conception of medicine given, until Hahnemann raised his eye to find the polar star of the *similia similibus curantur.* We, therefore, do not stand upon mere theory and speculation, but upon the safe foundation of experiment, experience and correct observations, called Induction. Likewise Hippocratic medicine has grown out of correct observations of what passed under the intelligent eye of the ancient physician. But it applied mostly to the physical condition of the organism, and brought physical and chemical substances to bear upon it. From this arose gradually through many centuries the sciences of physics and chemistry. But they have fulfilled their mission in regard to medicine, they have furnished the crude substances for Homœopathy, which on her part takes them in hand and potentiates them into something new and foreign to these two important branches of general science, into potencies, able to cure the sick and to ease the dying in their last moments. And this is owing to Hahnemann alone, the great messenger whom God sent to poor stricken humanity in order to relieve her sufferings and to restore the pristine vigor of man. For he was the first who made the action of medicine dependent upon that part of the human being which is indestructible and immortal. This is the very key-note and characteristic of Homœopathy, and must be firmly established in the mind of the homœopathician if he means to be one.

Hahnemann puts the calling of the physician into the duty of making sick people well, *i. e.*, of healing. People are sick whose condition deviates from the standard of health. Health is the normal oscillating equilibrium of the vital forces of the organism acting within certain limits, discerned by the observer in symptoms subjective and objective. Disease is the equilibrium of health disturbed with extension or contraction of the limits observed in symptoms subjective and objective. Disease is, therefore, not a thing, nor is health, they both are states of the organism changed either way and observable by the symptoms. In the language of Prof. A. B. Palmer (*North American Review*, March, 1882), "Diseases are phenomenal—are deviations from normal activities and normal compositions and structures in the organism." There is, therefore, no ostensible difference in the conception of disease among homœopathicians and allopathicians, for as such an one Dr. Palmer takes down Homœopathy and does not leave a good part in it. By using the convenient term allopathician, no offense is meant, because this term naturally grows out of the scientific question, how disease thus defined is to be met, and consequently rests upon a scientific justification.

Now, here is the important point where Hahnemann differs from all the physicians of past ages, though there have been such as had been on the right track. He lays it down as an incontrovertible fundamental fact, that "in health the spirit-like life-force (autocracy) which as dynamis animates the material body, rules absolutely and holds all its parts in admirably harmonic course of life in sensations and activities, in such a manner, that our in-dwelling rational spirit can use freely this living sound instrument for the higher ends of our being."—*Organon*, 5th ed., § 9.

This life-force or vital power which is the sum total of all the vital forces in the body, has, by late philosophers been thrown out, upon the ground of the development of physical and chemical science, but—*naturam expellas furca, tamen usque recurrit.* (You may throw out nature with the pitchfork, it will always return.) The idea of a vital power animating the body, will always hold its place, because the facts justify it; nay, every day's experience proves it, even to minds less cultivated than those of highly learned philosophers. They are right in so far, however, as it does not fit into the science of physics and chemistry, which has to deal exclusively with the nature and properties of matter. Even when the hypothetical atoms and molecules, the size of which has been calculated to a nicety, though nobody can furnish the proof—are called to assistance, they cannot help to explain the phenomena of the vital force which holds them in check and directs them, in order to serve for higher purposes. As soon as we have to deal with man, the scientific explorer must come to the conclusion that there is something governing the material body, and it might be called, for convenience sake, the spiritual body. But who governs the spiritual body? Partly the spirit, partly the soul, through the nervous system. There is a connecting link between the nervous system, the soul and the spirit, which has been called the nerve-spirit. "Through this nerve-spirit the soul is connected with the body and the body with the world. This nerve-spirit after death goes along with the soul and is indestructible. Through him the soul forms an ethereal envelope around the spirit. It is capable of growth after death, and by his instrumentality the inferior spirits produce sounds by which they can make themselves heard by men; they are, also, by his instrumentality, able to suspend gravity in the bodies, and to make themselves felt by men. A man, however, who dies in a perfectly pure and blessed state, which is very rare, does not take along this nerve-spirit after death; with those it remains, but also indestructible in the body, and afterwards after the general resurrection, when he joins the soul again, it forms the new ethereal body. Blessed spirits to whom this nerve-spirit does not adhere, cannot make themselves audible; this is mostly the doing of the unblessed spirits. The purer the soul of the departed one is becoming, the more it looses this nerve-spirit, which always returns to the earth."—*Kerner, dis Sebverin von Prevorst,* 3d ed., Stuttgart, Cotta, 1838, p. 187. This, then, is what Hahnemann calls the spirit-like vital power to which all the physical and chemical forces entering into the composition of the material body, are subservient, and its working is shown by the phenomena which life presents, and there is no other way to discern it than the outward symptoms of action, subjective and objective.

Now, Hahnemann contends, *Organon,* § 12, "only the morbidly tuned life-force brings forth the diseases," to which he adds the note: "How the vital force turns the organism to the morbid utterances, *i. e.,* how it produces disease, of this *how* the healing artist can never profit, and, therefore, it will be eternally hidden from him; only what was necessary for him to know of the disease, and what was fully sufficient for the sake of healing, has the Lord of life laid before his senses."

Diseases, therefore, are not of material but of spiritual origin, and must consequently be dealt with accordingly. It naturally also follows, that the whole complex of symptoms in a state of disease composes the true and only thinkable form of the disease present. How this complex is to be obtained is the office of the careful medical observer, and for that reason it is necessary that he should be a well-educated physician, well versed in all that pertains to medical and general science.

For that purpose it is not enough to count out the number of symptoms and cover them with the similar symptoms observed from the remedy, but he must, after doing that, digest them, find out in which relation they stand to each other, use all the necessary means to investigate the nature of changed secretion, temperature, gravity, moisture; he must consider the mental and emotional symptoms, how they may connect with the corporeal symptoms and so on. He, in short, must make a careful diagnosis of the case, to which purpose he must bring his knowledge of grammar, physics, chemistry, anatomy, pathology, pathological anatomy, physiology, psychology and all its auxiliaries, to bear upon it, and apply the instruments of investigation, such as the stethoscope, the plessimeter, the sphygmograph, the speculums, the ophthalmoscope, the laryngoscope, the odoscope, the endoscope, the thermometer, the chronoscope, the galvanoscope, etc. Only then the physician is capable of obtaining an intelligent picture of the complex of symptoms of disease constituting the true and only thinkable form of the disease in question.

Whoever does not accept these fundamental truths laid down by Hahnemann with a wonderful clearness and precision, would do better to spend his mental strength in other walks of life than in reviling this godly gift of Homœopathy with arguments which are not adequate to the purpose, because they start from premises entirely foreign to it.

Now, Hahnemann, having done with the notion of disease, proceeds to investigate how it is to be met in order to restore the organism to its healthy normal condition. And here we admire his acumen when, obtaining symptoms similar to chills and fever from the Peruvian bark, which he had taken for experiment, he fell upon the idea, that because the bark cures similar symptoms in the sick, it might be because in the healthy it produces similar symptoms. And thus he found by further experimentation, according to the rule of induction, that like cures like. Before all, Hippocrates taught it, but his expositor Galenus ignored it and put in its stead the baleful doctrine of *contraria contrariis.* Paracelsus taught it, and, after him, many others, down to the great Bichât, who declared emphatically as his principle: "*Similis organorum textura, similis functio, similis morbi, similis morborum exitus, similis therapia.*" *Similis therapia!* What else is the Hahnemannian Homœopathy if not *similis therapia?* But to none of all these men occurred the idea, that the simility of symptoms forms the very foundation of the healing art and science.

If this is so, then we must look for remedies which are able to reach this Hahnemannian life-force, this indestructible nerve-spirit being similar and proportionate to it, in the shortest, safest and pleasantest manner possible. They could not be found in the large doses of crude substances, as hitherto had been the fashion to apply; these would make too fearful inroads into the strength of the system and would waste too much substance of the organism, as the sad experience of the past teaches. It, therefore, on the basis of experience, occurred to him to lessen his doses by means of applying inert vehicles in order to attenuate them. Hard substances had to be treated by trituration with sugar of milk, and then, like the others, amenable to solution by water and alcohol, diluted further on by these fluids. In this manner he proceeded gradually at the hand of induction, trying and proving his remedies as he was going along, and found the process of potentiation, by which the medicinal forces of drugs and other substances are raised to an incredible degree, till he arrived at the fact that the thirtieth potency was sufficient to cure any curable, and some before thought incurable, acute and chronic disease, provided the remedy had been carefully selected according to symptoms-simility. What fault there can be found with such a judicious and scientific proceeding extending over the latter half of the lifetime of one of the most remarkable men who ever graced this globe for eighty-eight years, is not easily to be seen, and it is evident that whoever wants to make progress in the science and art of healing must travel by the same road to the goal set before him. All true and genuine homœopathicians have done it, and have not been disappointed, as the record of their labors shows, and the general good name they possess in the hearts of the people.

### IV.

From all this follows that, if a large dose cures, it is owing to the homœopathicity of the drug and not to the large size of the dose, and it proceeds from the action of those few infinitesimals which make their impression upon the nervous centre, or upon the nerves directly, mediating the restoration of health by their hygiopoetic or healing power over the parts submitted to their influence, whilst the large bulk is carried off by the sewerage of the body or made inert by envelopment, and harm thus is prevented from an injurious action of the mass upon the system.

But it also follows that crude drugs are rendered homœopathic by potentiation, if they do exert either too much, or none, or too little medicinal action upon the organism, and homœopathic means able to cure a similar suffering.

To use such remedies technically called high potencies in order to make sick people well, is the true calling of the physician, according to Hahnemann.—*Organon*, § 1.

